# Effects of major depression and bipolar disorder on erectile dysfunction: a two-sample mendelian randomization study

**DOI:** 10.1186/s12920-023-01498-8

**Published:** 2023-03-30

**Authors:** Wei-Kang Chen, Tao Zhou, Dong-Dong Yu, Jing-Ping Li, Jing-Gen Wu, Le-Jun Li, Zhong-Yan Liang, Feng-Bin Zhang

**Affiliations:** 1 Department of Reproductive Endocrinology, Women’s Hospital, School of Medicine, Zhejiang University, Hangzhou, China; 2 Department of Urology, The First Affiliated Hospital of Wenzhou Medical University, Wenzhou, China; 3 Department of Urology, Huzhou Central Hospital, Affiliated Central Hospital Huzhou University, Huzhou, China

**Keywords:** Mendelian randomization, Major depression, Bipolar disorder, Erectile dysfunction, Causal association

## Abstract

**Background and Aims:**

There are currently no clear conclusions about whether major depression (MD) and bipolar disorder (BD) increase the risk of erectile dysfunction (ED). In our study, we used a Mendelian randomization (MR) analysis to discover the causal associations between MD, BD and ED.

**Methods:**

We got single-nucleotide polymorphisms (SNPs) related to MD, BD and ED from the MRC IEU Open genome-wide association study (GWAS) datasets. After a series of selection, SNPs left were selected as instrumental variables (IVs) of MD and BD for the following MR test to evaluate the relationship of genetically predicted MD or BD with the incidence of ED. Among them, we used the random-effects inverse-variance weighted (IVW) method as the main analysis. Finally, sensitivity analyses were further performed using Cochran’s Q test, funnel plots, MR-Egger regression, Leave-one-out method and MR- pleiotropy residual sum and outlier (PRESSO).

**Results:**

Genetically-predicted MD was causally related to the incidence of ED in the IVW methods (odds ratio (OR), 1.53; 95% confidence interval (CI), 1.19–1.96; p = 0.001), while no causal impact of BD on the risk of ED (OR = 0.95, 95% CI 0.87–1.04; p = 0.306). The results of sensitivity analyses supported our conclusion, and no directional pleiotropy were found.

**Conclusion:**

The findings of this research found evidence of a causal relationship between MD and ED. However, we did not find a causal relationship between BD and ED in European populations.

**Supplementary Information:**

The online version contains supplementary material available at 10.1186/s12920-023-01498-8.

## Introduction

Erectile dysfunction (ED) is an important part of sexual dysfunction and can cause a decrease in the life quality of the patient and his partner. From the National Institutes of Health, the most commonly cited definition of ED is the inability to obtain and maintain an erection for satisfactory sexual intercourse firm enough [1]. The European Association of Urology (EAU) 2021 Andrology Disease Guide indicated that the incidence of ED increased with age, ranging from 12 to 82.9% [2]. Although ED can be considered a vascular disease essentially, it is also closely related to neurological and mental health. For example, several studies report that ED is commonly found in some men with mental illness, including major depression (MD) [3], anxiety [4, 5] and schizophrenia [6].

In the 2017 Global Burden of Disease Study [7], depression is the third cause of non-fatal health loss, and affect over 300 million people worldwide [8]. According to previous literature, patients with ED often have MD [9, 10] with a frequency ranging from 8.7%14 to 43.1% [11]. Recently, a Meta-analysis reported that depression may lead to ED (OR = 1.39, 95% CI: 1.35-42) [12], has further substantiated the association between MD and ED.

Bipolar disorder (BD), always characterized by irritability or euphoria or elation and increased energy activity or levels, is a serious mental illness. Globally, the lifetime incidences of BD type I and type II are approximately 0.6% and 0.4%, however this value in developed countries is higher [13, 14]. BD is related to significant functional impairment, a higher rate of suicide, lower quality of life and a likelihood of high comorbidity. There is currently a lack of literature describing the relationship between BD and ED, but the recently cohort study of Hou et al. [15] found that BD patients had a higher prevalence of ED than controls, attracting public attention.

To the best of our knowledge, extant studies are largely based on observational epidemiological designs and are therefore susceptible to reverse causality and unmeasured confounding factors [16]. To avoid this situation, Mendelian randomization (MR) has the advantage of using genetic variation as an instrumental variable, addressing observational research bias, thereby providing an alternative approach to explore causality [17, 18]. In the study, we used a MR approach to investigate the causal relationship between MD and BD on the risk of developing ED.

## Materials and methods

### Study design

We used a two-sample MR design to detect the potential causal association of MD and BD on the risk of ED. The hypothesis of the MR study consist of three conditions: (i) the instrumental variants (IVs) should be associated with exposures of MD and BD; (ii) No clear correlation between IVs and the confounders; (iii) IVs have an effect on risk of ED only through the exposure of interest (MD or BD) and not through other means [19]. Only when all three of these conditions are met can the MR design reverse causality, control for potential confounders and provide robust estimates of causal effects [20]. Data on the associations of single-nucleotide polymorphisms (SNPs) with MD, BD and ED were obtained from publicly available large-scale genome-wide association studies (GWAS) [21–23], which could be downloaded from the MRC IEU Open GWAS datasets (Supplementary Table 1). The summary statistics of MD (GWAS ID: ieu-b-102) were obtained from Psychiatric Genomics Consortium (PGC) and the UK Biobank (UKB), extracted from 170,756 cases and 329,443 controls based on European samples; the summary statistics of BD (GWAS ID: ieu-b-41) were obtained from the PGC, including 13,413,244 SNPs of 170,756 European cases and 329,443 European controls; the summary statistics of ED (GWAS ID: ebi-a-GCST006956) were extracted from the UKB and the Estonian Genome Center of the University of Tartu (EGCUT), which were obtained from 223,805 European Samples.

All the data from MR are publicly accessible (https://gwas.mrcieu.ac.uk/; last accessed on September 7, 2022). Ethical approval were waived for this research, and all subjects in the original GWAS have obtained informed consent.

### Selection of genetic variants

In this study, we obtained SNPs that are significantly related to MD (p < 5 × 10^− 8^) from GWAS summary data [24, 25], while we relaxed the GWAS p-value threshold to 5 × 10^− 7^ in BD in order to obtain a suitable number of SNPs for subsequent analysis [26]. Then, we used the PLINK clumping method to calculate the linkage disequilibrium (LD) through the two-sample MR package and selected independent SNPs with the following conditions (R^2^ < 0.001, window size = 10,000 kb) [27], to ensure that all the left IVs for MD and BD are not in LD. We estimate the strength of the IVs on the basis of the F statistic. The formula is as follows: F = R^2^(N-2) (1-R^2^) (R^2^: variance of exposure explained by selected instrumental variables; N:sample size) [28]; R^2^ = 2×EAF×(1-EAF)×beta^2/((2×EAF×(1-EAF)×beta^2) + 2×EAF×(1-EAF)×se×N×beta^2) (beta: effect size for SNP; se: standard error for SNP; N:sample size) [29]. IVs were selected whose F > 10. After harmonizing the SNPs in the data source by effector alleles [30], we discovered each instrument SNP in the PhenoScanner GWAS database [31] to assess any prior association (P < 5 × 10^− 8^) with possible confounding factors (that is sleeplessness or insomnia, body mass index, smoking status, education, hematocrit, cardiovascular diseases, et al.) [32–34] to avoid potential confounding. Finally, the SNPs left were selected as IVs for the following MR test.

### Statistical analysis

In the study, we applied the random-effects inverse-variance weighted (IVW) method as the main analysis to evaluate the casual relation of genetically predicted MD and BD with the risk of ED [35]. Other methods including MR Egger [36], weighted-median [37], weighted mode [38] and simple mode [39] were also applied. The main principles are as follows: (1) In the absence of heterogeneity and pleiotropy, the estimation results of IVW are preferred;(2) When there is only heterogeneity and no pleiotropy, the results of Weighted Medium method are preferred (the random effect model of IVW can also be used);(3) When there is multiple validity, the results calculated by MR Egger method are preferred [40]. Besides, several sensitivity analyses were carried out to evaluate the strength of the association. First, Cochran’s Q test and funnel plots were performed to assess the heterogeneity [41]. Second, we applied MR-Egger regression to recognize the existence of directional pleiotropy by calculating whether the intercept was statistically away from zero [36]. Third, we used the Leave-one-out method to verify the robustness of the findings [42]. Fourth, in order to detect possible outliers, we apply the MR pleiotropy residual sum and outlier (MR-PRESSO) test [43].

We used odds ratios (ORs) with their 95% confidence intervals (CIs) to present the associations between MD and BD and risk of ED and applied RStudio (version 2022.02.3) with ‘TwoSampleMR’ and ‘MR-PRESSO’ to perform MR analyses. In this study, p < 0.05 was considered a statistically significant difference.

## Results

### Genetically predicted MD on ED

**Fig. 1:**
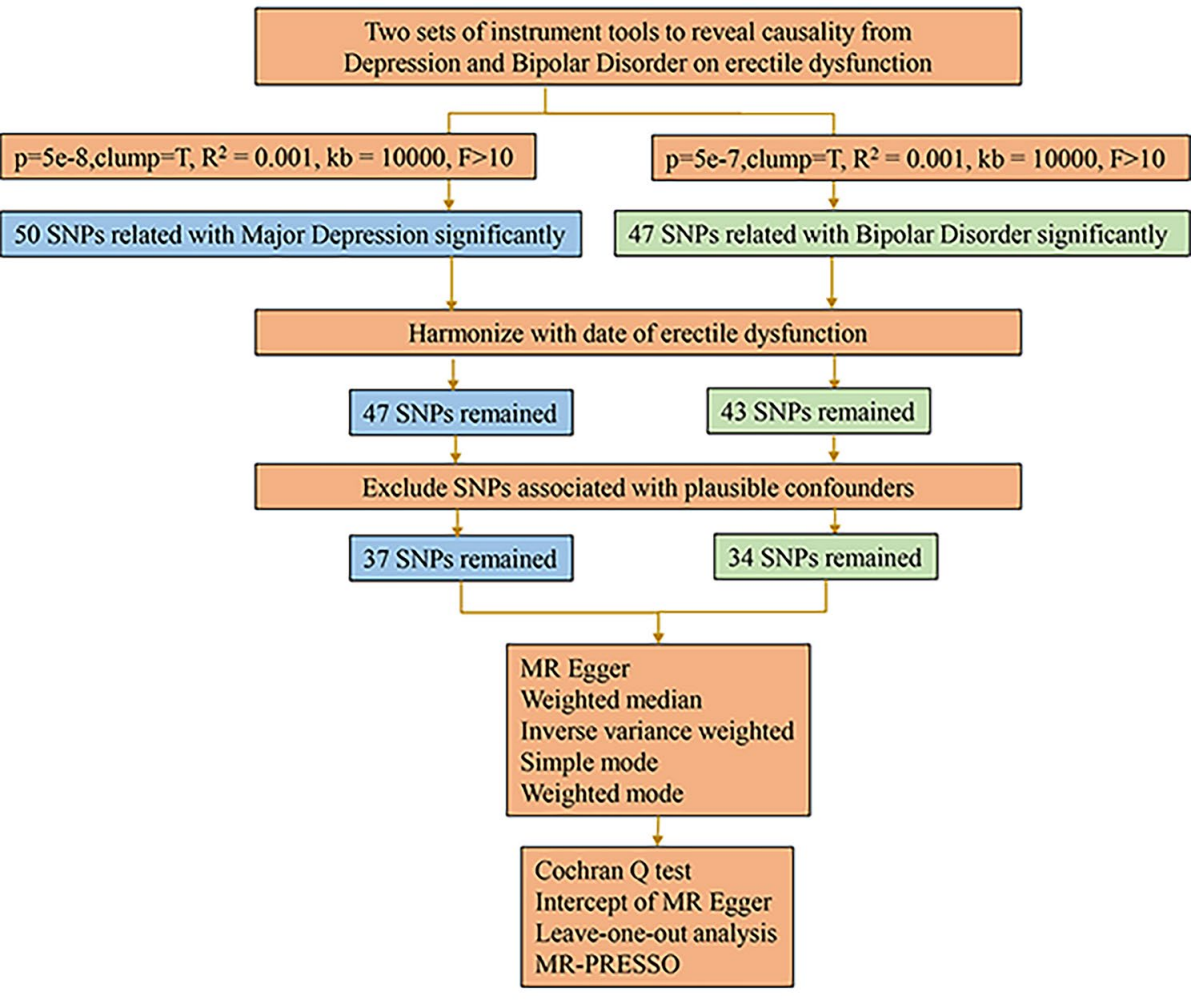
Workflow of MR study revealing causality from MD and BD on ED.

After the above selection (the specific flow chart is shown in Fig. 1), 37 IVs were left, accounting for approximately 24.4% of the observed variance of MD and all the F-statistics were above 10, ranging from 339.5 to 86003.0 (Supplementary Table 2).

**Fig. 2:**
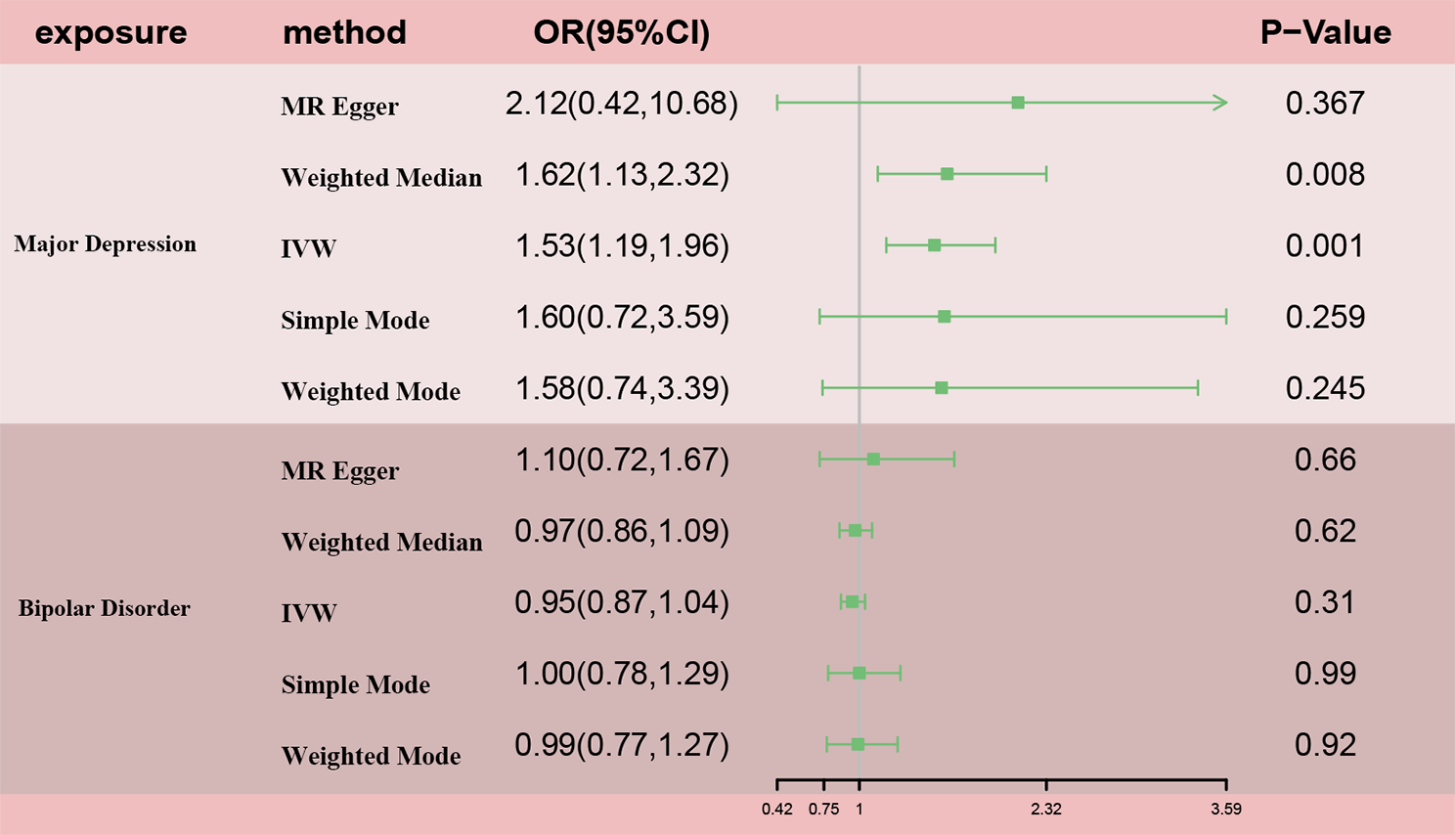
OR plot for MD and BD.

**Fig. 3:**
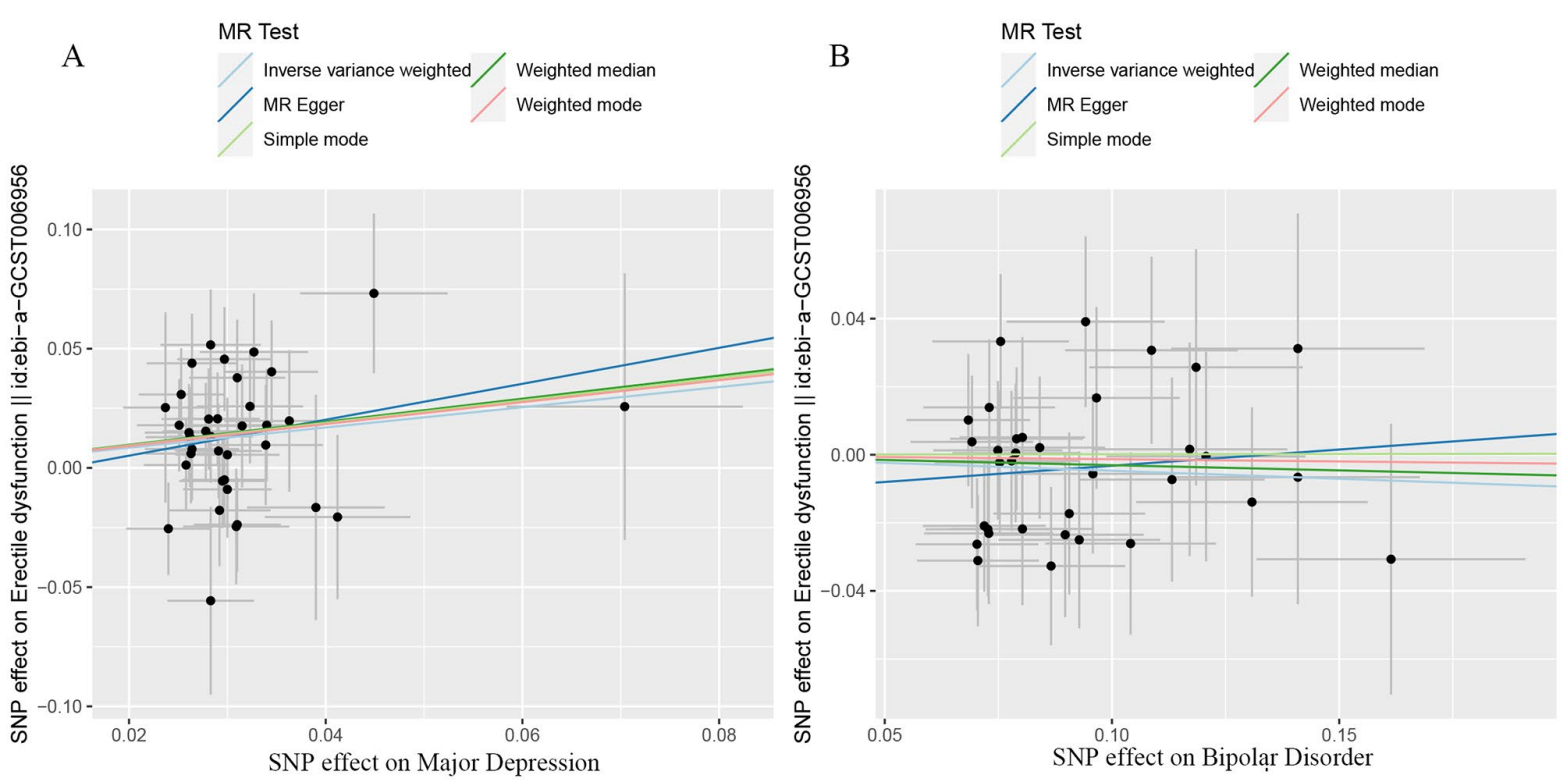
The causality of MD(A) and BD(B) on ED risk. The slope represents the magnitude of the causal effect

**Fig. 4:**
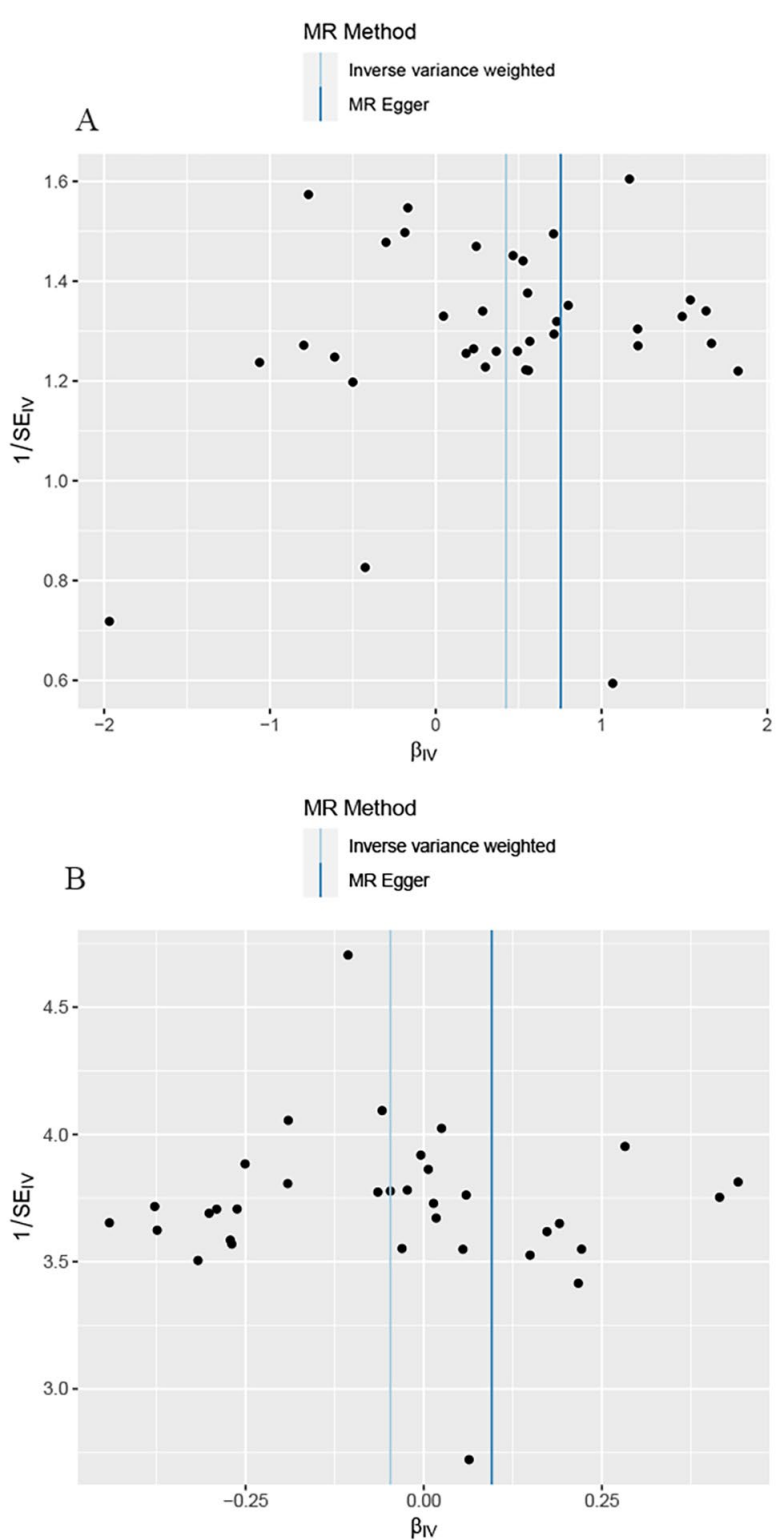
Funnel plot to assess the heterogeneity of MD(A) and BD(B).

**Fig. 5:**
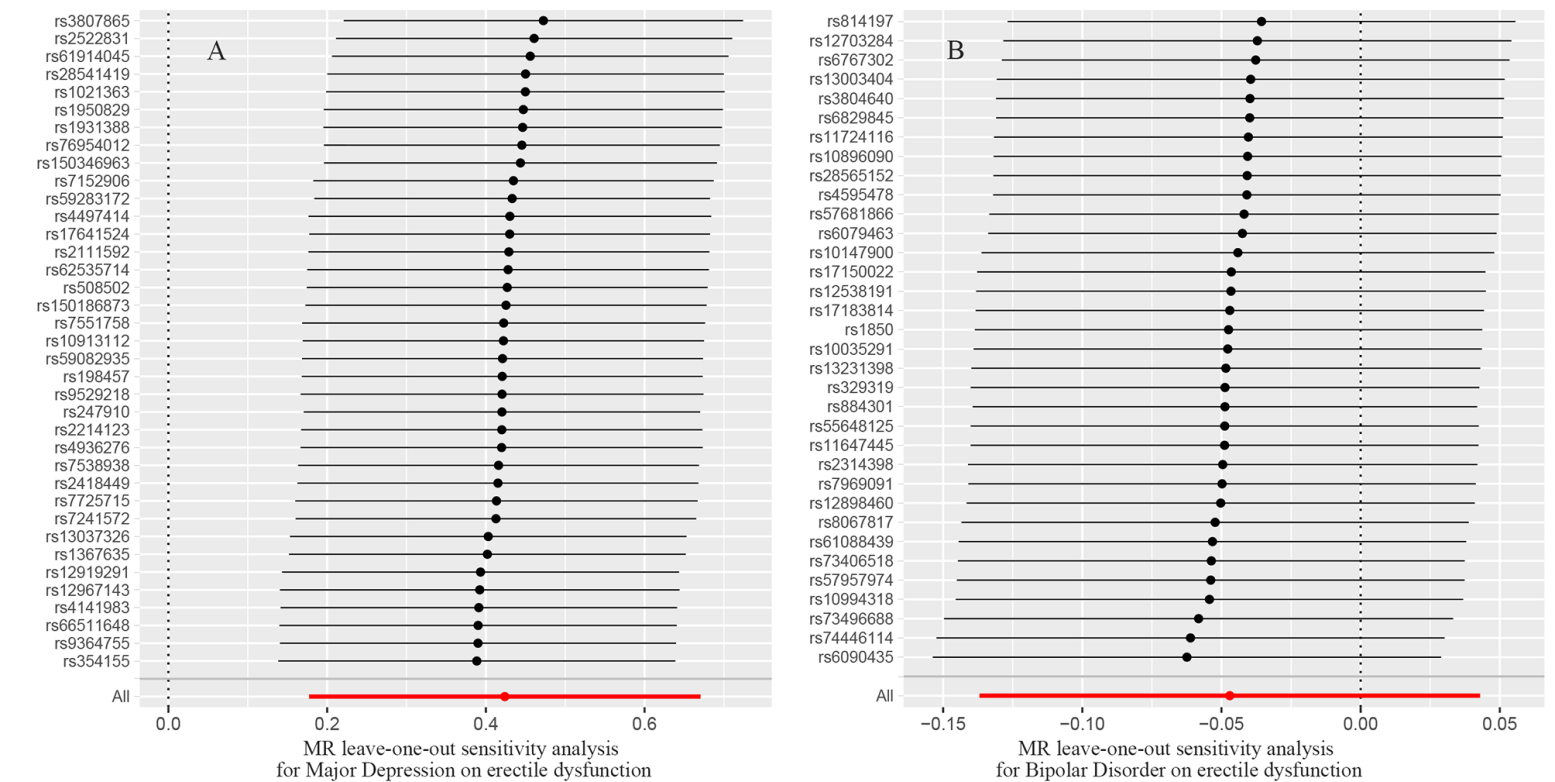
Leave-one-out analysis of the effect of MD(A) and BD(B) on ED.

Genetically predicted MD was related to higher odds of ED (OR = 1.53, 95% CI 1.19–1.96; p = 0.001) in the IVW analyses (Figs. 2 and 3A). Meanwhile, similar results were discovered by weighted median approaches (OR = 1.622, 95% CI = 1.13–2.32, p = 0.008), weighted mode approaches (OR = 1.58, 95% CI = 0.74–3.39, p = 0.245), simple mode (OR = 1.60, 95% CI = 0.72–3.59, p = 0.259) and MR-Egger regression (OR = 2.12, 95% CI = 0.42–10.68, p = 0.367) (Fig. 2). No heterogeneity was found in the study with a Cochran Q-test (P = 0.436 of MR-Egger; p = 0.475 of IVW) (Table 1) and funnel plots (Fig. 4A). The MR-Egger intercept did not deviate significantly from zero with a p-value of 0.688 (Table 1). The leave-one-out test found that no significant differences was discovered while we removed a single SNP and applied the MR analysis again, demonstrating our results’ robustness (Fig. 5A). By using the MR-PRESSO test, Outliers are not found, verifying the absence of unknown pleiotropic effects of the genetic instruments. After calculation, we found the MR analyses of MD had 100% statistical power.

**Table 1:** Pleiotropy tests and heterogeneity of MR

	Pleiotropy test			Heterogeneity test					
				MR Egger			IVW		
	egger_intercept	se	pval	Q	Q_df	Q_pval	Q	Q_df	Q_pval
**MD**	-0.010	0.025	0.688	35.694	35	0.436	35.861	36	0.475
**BD**	-0.013	0.019	0.500	22.777	32	0.885	23.242	33	0.896

Abbreviations: MD: Major depression; BD: Bipolar Disorder; IVW, inverse variance weighted; MR, Mendelian randomization.

### Genetically predicted BD on ED

After the above selection (the specific flow chart is shown in Fig. 1), 34 IVs were left, accounting for approximately 3.9% of the observed variance of BD (the F-statistics range from 33.8 to 74.6) (Supplementary Table 3). Genetically predicted BD was not related to ED (OR = 0.95, 95% CI 0.87–1.04; p = 0.306) in the IVW analyses (Figs. 2 and 3B). The consistent results were obtained in the weighted median approaches (OR = 0.97, 95% CI = 0.86–1.09, p = 0.617), weighted mode approaches (OR = 0.99, 95% CI = 0.77–1.27, p = 0.920), simple mode (OR = 1.00, 95% CI = 0.78–1.29, p = 0.991) and MR-Egger regression (OR = 1.10, 95% CI = 0.72–1.67, p = 0.659) (Fig. 2). There was no heterogeneity found by a Cochran Q-test (P = 0.885 of MR-Egger; p = 0.896 of IVW) (Table 1) and funnel plots (Fig. 4B). The MR-Egger intercept did not deviate significantly from zero with a p-value of 0.896 (Table 1). The leave-one-out test showed that there were no significant differences (Fig. 5B) and the MR-PRESSO test did not find any outliers. However, statistical power failed to reach 80% to discovery the weak associations.

## Discussion

In the study, we used a MR approach to investigate the causal relationship between MD or BD on the risk of ED. The findings of this research found evidence of a causal relationship between MD and ED. However, we did not find a causal relationship between BD and ED.

As far as we know, the relationship between depression and ED is currently unclear. Some scholars point out that depression can increases the risk of ED [44, 45], while others do not agree [46].

A recent meta-analysis indicated an association between depression and ED, which the overall OR for studies evaluating depression exposure and risk of ED was 1.39 (95% CI: 1.35–1.42) [12]. Since OR acts as an association measure, it can only prove the existence of an association. Therefore, the above study cannot clarify the causal relationship between ED and depression and its direction. In our study, we took advantage of MR, a better study design method, which is free from bias and can accurately reveal causal relationships. Recently, a newly published article also used MR method, and further confirmed that MD plays a potentially causal role in the occurrence of ED [47]. In their study, they used the data of three institutions (PGC, the UKB and 23andMe) and did not remove possible confounding factors. However, in our study, we selected the data of two consortiums (PGC and UKB), considering the reliability of data sources and the potential overlap of data between consortiums. In addition, we discovered each instrument SNP in the PhenoScanner GWAS database to assess any prior association with possible confounding factors to avoid potential confounding. Finally, we also found that MD could increase the risk of ED with the OR was 1.53 (95% CI 1.19–1.96). Our findings further clarify the impact of MD on ED and provide more evidence for clinical practice.

The mechanisms underlying how MD leads to ED remain to be elucidated and established [3]. However, some scholars have proposed relevant behavioral and biological models to explain the mechanism of the increased risk of ED in patients with depression [48]. Makhlouf et al. suggest that depressed patients often exhibit a lack of confidence and negative thinking, which in turn leads to decreased erectile function [48]. Biological models suggest the hypothalamic-pituitary-adrenal (HPA) axis is affected by depression, resulting in a high production of catecholamines, which in turn causes cavernosal muscle dysregulation and ED [49]. Depression may inhibit the activity of parasympathetic nerves, thereby decreasing the inflow of blood to the penis and inhibiting penile smooth muscle relaxation [50]. Moreover, most antidepressants have also been found to have some adverse effect on erectile function [50]. Depending on the various drugs, the incidence of ED may range from 25.8 to 80.3% [51]. Unfortunately, ED may persist after selective serotonin reuptake inhibitors (SSRIs) are discontinued, with this treacherous condition being only recently defined as post-SSRI sexual dysfunction [52, 53]. Besides, studies have found that depressed patients have lower levels of testosterone than non-depressed patients, and low testosterone is thought to be associated with ED [54, 55].

As for BD, there was currently a lack of literature describing the relationship between BD and ED. Recently, Hou et al. found that the incidence of ED in BD patients was significantly higher than that in the control group (HR = 2.24, 95% CI: 1.71–2.94) [15], through a research of 5,150 BD male patients in the Taiwan’s National Health Insurance. The specific relevant mechanism may be as follows. In clinical practice, BD is primarily treated with antipsychotics, mood stabilizers, and antidepressants, which have been found to cause ED. In addition, a large proportion of BD patients are accompanied by sleep disturbances, which in turn reduce testosterone levels in men and cause and lead to sexual dysfunction. Considering the above potential mechanism and the potential causal relationship between MD and ED we discovered, our team assume that BD can also increase the risk of ED and made effort to study the causal relationship between BD and ED by using MR method. However, based on our study, we did not find the clear evidence that BD has a direct contribution to the risk of ED. Therefore, it suggests that further research is needed on the relationship between BD and ED.

## Strengths and limitations

The MR study design is one of the greatest strengths of this study. This approach can reverse causality inherent and minimize residual confounding in observational studies. Besides, it can allow us to discovery potential causal relationships between ED and MD or BD. The study can further support the results through other secondary analytical approaches and sensitivity analyses, increasing the reliability of our conclusions. In addition, we extracted the instrumental variables from the most recent GWAS available with confidence to minimize weak instrumental bias.

However, there were some several limitations. First, the data from GWASs of this study came from European, so that the similar study should be investigated in other populations. Second, there are different subtypes of ED (non-vasculogenic or vasculogenic), MD and BD, which were not distinguished in this study. Subsequent studies could be devoted to ED analysis of different subgroups. Thirdly, only 3.9% of the observed variance in BD was explained by IVs, so the statistical power may be insufficient. Therefore, for this negative result, we need to interpret it with caution to avoid drawing this conclusion due to insufficient power.

## Conclusion

The findings of this research found evidence of a causal relationship between MD and ED. But the mechanism of the association between MD and ED remains to be discovered. On the other hand, we did not find a causal relationship between BD and ED in European populations, which need further in-depth research to verify.

## Electronic supplementary material

Below is the link to the electronic supplementary material.

Supplementary Material 1

## Data Availability

The datasets generated and analysed during the current study are available in the IEU open gwas project [https://gwas.mrcieu.ac.uk/], and the GWAS ID are ieu-b-102, ieu-b-41and ebi-a-GCST006956, respectively.
