# Supplementary material for: Effects of major depression and bipolar disorder on erectile dysfunction: a two-sample mendelian randomization study
Source: BMC Med Genomics. 2023 Mar 30;16:66. doi: 10.1186/s12920-023-01498-8 (PMC10061895; doi:10.1186/s12920-023-01498-8)
Supplement: Supplementary file 1 — Supplementary Material 1 [file 12920_2023_1498_MOESM1_ESM.docx]

| Supplementary table 1: Details of the GWASs included in the Mendelian randomization | | | | | |  |  |  |  |  |  |  |  |
| --- | --- | --- | --- | --- | --- | --- | --- | --- | --- | --- | --- | --- | --- |
|  |  |  |  |  |  |  |  |  |  |  |  |  |  |
| **GWAS ID** | **Trait** | **Consortium** | **Population** | **Participants** | **PMID** |  |  |  |  |  |  |  |  |
| **ieu-b-102** | Major depression | UKB and PGC | European | 500199 | 30718901 |  |  |  |  |  |  |  |  |
| **ieu-b-41** | Bipolar Disorder | PGC | European | 51,710 | 31043756 |  |  |  |  |  |  |  |  |
| **ebi-a-GCST006956** | Erectile dysfunction | UKB, EGCUT and PHB | European | 223,805 | 30583798 |  |  |  |  |  |  |  |  |
|  |  |  |  |  |  |  |  |  |  |  |  |  |  |
| Abbreviations: UKB, UK Biobank; PGC, Psychiatric Genomics Consortium; EGCUT,  the Estonian Genome Center of the University of Tartu; PHB, hospital-recruited Partners HealthCare Biobank. | | | | | | | | | | | | | |

Supplementary table 2: instrumental variables for major depression.

| **SNP** | **chr** | **positon** | **EA** | **OA** | **EAF** | **beta** | **se** | **p** | **R^2** | **F** |
| --- | --- | --- | --- | --- | --- | --- | --- | --- | --- | --- |
| **rs7551758** | 1 | 52274078 | G | T | 0.53 | 0.03 | 0.0043 | 5.11E-11 | 0.00138 | 690.48 |
| **rs10913112** | 1 | 175913828 | T | C | 0.38 | -0.03 | 0.0045 | 4.53E-09 | 0.00106 | 530.72 |
| **rs17641524** | 1 | 197704717 | T | C | 0.21 | -0.03 | 0.0053 | 1.5E-08 | 0.00215 | 1075.44 |
| **rs354155** | 1 | 49675276 | C | G | 0.09 | -0.04 | 0.0075 | 1.75E-09 | 0.01522 | 7728.85 |
| **rs7538938** | 1 | 67132262 | C | T | 0.56 | 0.03 | 0.0043 | 7.29E-09 | 0.00085 | 427.11 |
| **rs4141983** | 1 | 18122009 | C | T | 0.33 | -0.03 | 0.0046 | 9.69E-09 | 0.00112 | 559.29 |
| **rs2111592** | 2 | 208049581 | A | G | 0.31 | 0.03 | 0.0046 | 1.35E-08 | 0.00110 | 550.86 |
| **rs66511648** | 3 | 117515519 | C | T | 0.28 | 0.03 | 0.0048 | 6.03E-10 | 0.00187 | 935.36 |
| **rs76954012** | 3 | 115977242 | A | T | 0.09 | 0.04 | 0.0074 | 2.41E-08 | 0.01065 | 5382.90 |
| **rs247910** | 5 | 87630769 | G | A | 0.46 | 0.02 | 0.0043 | 4.71E-08 | 0.00068 | 339.47 |
| **rs7725715** | 5 | 164487555 | A | G | 0.53 | 0.03 | 0.0043 | 1.61E-11 | 0.00152 | 761.45 |
| **rs150186873** | 6 | 27182377 | C | A | 0.03 | 0.07 | 0.012 | 4.51E-09 | 0.14671 | 86002.95 |
| **rs9364755** | 6 | 165117329 | G | A | 0.23 | 0.03 | 0.0051 | 3.49E-08 | 0.00163 | 819.15 |
| **rs2214123** | 6 | 67000001 | G | A | 0.65 | -0.03 | 0.0045 | 8.56E-09 | 0.00104 | 522.66 |
| **rs2522831** | 7 | 82448100 | C | T | 0.47 | 0.02 | 0.0043 | 2.11E-08 | 0.00071 | 356.99 |
| **rs150346963** | 7 | 117625599 | T | C | 0.41 | 0.03 | 0.0044 | 1.16E-10 | 0.00141 | 706.56 |
| **rs3807865** | 7 | 12250402 | A | G | 0.41 | 0.03 | 0.0044 | 1.09E-12 | 0.00203 | 1017.77 |
| **rs59082935** | 7 | 38724868 | T | C | 0.13 | 0.04 | 0.0066 | 3.07E-08 | 0.00572 | 2879.89 |
| **rs62535714** | 9 | 37182655 | A | G | 0.16 | 0.03 | 0.0058 | 4.69E-09 | 0.00383 | 1921.66 |
| **rs1931388** | 9 | 11203149 | G | A | 0.40 | -0.03 | 0.0044 | 1.68E-11 | 0.00167 | 834.39 |
| **rs59283172** | 9 | 25232978 | A | G | 0.11 | -0.04 | 0.007 | 2.41E-08 | 0.00809 | 4078.56 |
| **rs2418449** | 9 | 119731359 | C | T | 0.28 | -0.03 | 0.0048 | 4.25E-09 | 0.00150 | 749.30 |
| **rs1021363** | 10 | 106610839 | G | A | 0.64 | -0.03 | 0.0045 | 2.29E-11 | 0.00182 | 912.81 |
| **rs198457** | 11 | 61471678 | T | C | 0.19 | -0.03 | 0.0056 | 1.9E-08 | 0.00276 | 1381.92 |
| **rs4497414** | 11 | 88756779 | C | T | 0.44 | 0.03 | 0.0044 | 2.93E-11 | 0.00158 | 790.00 |
| **rs4936276** | 11 | 113365141 | C | G | 0.62 | 0.03 | 0.0044 | 3.57E-10 | 0.00131 | 657.89 |
| **rs61914045** | 12 | 52352301 | A | G | 0.20 | 0.03 | 0.0054 | 7.96E-09 | 0.00246 | 1233.58 |
| **rs9529218** | 13 | 31790053 | T | C | 0.20 | -0.03 | 0.0054 | 2.23E-10 | 0.00361 | 1809.92 |
| **rs508502** | 13 | 80921519 | T | C | 0.30 | -0.03 | 0.0048 | 3.56E-08 | 0.00117 | 583.64 |
| **rs1950829** | 14 | 42097937 | G | A | 0.52 | -0.03 | 0.0043 | 4.74E-12 | 0.00167 | 837.76 |
| **rs7152906** | 14 | 75125540 | C | T | 0.52 | 0.03 | 0.0043 | 1.87E-09 | 0.00095 | 476.82 |
| **rs28541419** | 15 | 88945878 | G | C | 0.23 | -0.03 | 0.0052 | 1.76E-08 | 0.00189 | 946.83 |
| **rs12919291** | 16 | 13800430 | C | G | 0.19 | 0.03 | 0.0055 | 3.09E-09 | 0.00314 | 1576.67 |
| **rs12967143** | 18 | 53099012 | C | G | 0.70 | -0.03 | 0.0047 | 2.53E-13 | 0.00333 | 1669.51 |
| **rs7241572** | 18 | 77580712 | A | G | 0.20 | 0.03 | 0.0054 | 2.43E-09 | 0.00294 | 1473.37 |
| **rs1367635** | 18 | 50861409 | C | T | 0.51 | 0.03 | 0.0043 | 4.35E-09 | 0.00088 | 440.90 |
| **rs13037326** | 20 | 44692598 | T | C | 0.26 | 0.03 | 0.0049 | 2.4E-10 | 0.00226 | 1133.69 |

Abbreviations: chr, chromosome; EA, effect allele; OA, other allele; EAF, effect allele frequency.

Supplementary table 3: instrumental variables for Bipolar Disorder.

| **SNP** | **chr** | **positon** | **EA** | **OA** | **EAF** | **beta** | **se** | **p** | **R^2** | **F** |
| --- | --- | --- | --- | --- | --- | --- | --- | --- | --- | --- |
| **rs57681866** | 2 | 57975714 | A | G | 0.06 | -0.16 | 0.0296 | 5.001E-08 | 0.00065 | 33.78 |
| **rs10994318** | 10 | 62125856 | C | G | 0.06 | 0.14 | 0.0279 | 4.494E-07 | 0.00069 | 35.84 |
| **rs17183814** | 2 | 166152389 | A | G | 0.07 | -0.14 | 0.0268 | 1.49E-07 | 0.00072 | 37.31 |
| **rs10147900** | 14 | 105092031 | T | C | 0.14 | 0.13 | 0.0255 | 3.033E-07 | 0.00076 | 39.21 |
| **rs73406518** | 15 | 42847033 | T | C | 0.09 | 0.12 | 0.0235 | 4.593E-07 | 0.00082 | 42.55 |
| **rs13231398** | 7 | 110197412 | C | G | 0.11 | -0.12 | 0.0219 | 3.361E-08 | 0.00088 | 45.66 |
| **rs55648125** | 6 | 50816718 | G | A | 0.11 | 0.12 | 0.0215 | 4.922E-08 | 0.00090 | 46.51 |
| **rs17150022** | 7 | 24771777 | C | T | 0.12 | 0.11 | 0.0204 | 2.701E-08 | 0.00095 | 49.02 |
| **rs73496688** | 11 | 79156748 | A | T | 0.15 | 0.11 | 0.019 | 1.047E-08 | 0.00102 | 52.63 |
| **rs11724116** | 4 | 162294038 | T | C | 0.16 | -0.10 | 0.0188 | 3.267E-08 | 0.00103 | 53.19 |
| **rs61088439** | 8 | 111137092 | A | T | 0.16 | 0.10 | 0.0183 | 1.337E-07 | 0.00106 | 54.64 |
| **rs12538191** | 7 | 44980824 | A | G | 0.24 | -0.10 | 0.0182 | 1.461E-07 | 0.00106 | 54.94 |
| **rs4595478** | 10 | 111800145 | C | T | 0.83 | -0.09 | 0.0178 | 1.919E-07 | 0.00109 | 56.18 |
| **rs74446114** | 2 | 28066122 | C | T | 0.19 | 0.09 | 0.0174 | 6.235E-08 | 0.00111 | 57.47 |
| **rs10896090** | 11 | 65945186 | G | A | 0.19 | -0.09 | 0.0173 | 2.083E-07 | 0.00112 | 57.80 |
| **rs6079463** | 20 | 14475757 | G | A | 0.20 | 0.09 | 0.0167 | 5.378E-08 | 0.00116 | 59.88 |
| **rs12703284** | 7 | 140767992 | G | C | 0.78 | 0.09 | 0.0163 | 9.813E-08 | 0.00119 | 61.35 |
| **rs28565152** | 5 | 7542911 | A | G | 0.25 | 0.08 | 0.0158 | 3.83E-07 | 0.00122 | 63.29 |
| **rs6090435** | 20 | 62132711 | A | G | 0.50 | -0.08 | 0.015 | 4.393E-07 | 0.00129 | 66.66 |
| **rs10035291** | 5 | 80796368 | C | T | 0.32 | -0.08 | 0.0147 | 1.147E-07 | 0.00131 | 68.02 |
| **rs12898460** | 15 | 38986813 | T | C | 0.30 | -0.08 | 0.0146 | 6.354E-08 | 0.00132 | 68.49 |
| **rs57957974** | 8 | 145076529 | A | C | 0.36 | 0.07 | 0.0145 | 4.977E-07 | 0.00133 | 68.96 |
| **rs2314398** | 2 | 97413488 | G | C | 0.32 | -0.08 | 0.0144 | 5.92E-09 | 0.00134 | 69.44 |
| **rs1850** | 20 | 49620271 | C | T | 0.35 | 0.08 | 0.0143 | 1.49E-07 | 0.00135 | 69.93 |
| **rs11647445** | 16 | 9926966 | G | T | 0.35 | 0.07 | 0.0142 | 1.217E-07 | 0.00136 | 70.42 |
| **rs6829845** | 4 | 123510766 | G | A | 0.67 | -0.07 | 0.0142 | 2.94E-07 | 0.00136 | 70.42 |
| **rs329319** | 5 | 133906609 | G | A | 0.57 | -0.08 | 0.0139 | 1.539E-08 | 0.00139 | 71.94 |
| **rs884301** | 17 | 53367464 | T | C | 0.38 | 0.08 | 0.0138 | 5.801E-09 | 0.00140 | 72.46 |
| **rs13003404** | 2 | 194464707 | C | T | 0.59 | 0.07 | 0.0137 | 1.176E-07 | 0.00141 | 72.99 |
| **rs8067817** | 17 | 38163936 | T | C | 0.56 | 0.07 | 0.0136 | 4.503E-07 | 0.00142 | 73.53 |
| **rs3804640** | 3 | 107793709 | G | A | 0.46 | -0.07 | 0.0135 | 9.265E-08 | 0.00143 | 74.07 |
| **rs6767302** | 3 | 141582961 | G | A | 0.50 | -0.07 | 0.0135 | 2.123E-07 | 0.00143 | 74.07 |
| **rs7969091** | 12 | 49464449 | G | A | 0.44 | 0.07 | 0.0135 | 3.248E-07 | 0.00143 | 74.07 |
| **rs814197** | 1 | 61092456 | G | T | 0.46 | 0.07 | 0.0134 | 1.299E-07 | 0.00144 | 74.62 |

Abbreviations: chr, chromosome; EA, effect allele; OA, other allele; EAF, effect allele frequency.
